# Differential transgene expression patterns in Alzheimer mouse models revealed by novel human amyloid precursor protein‐specific antibodies

**DOI:** 10.1111/acel.12508

**Published:** 2016-07-29

**Authors:** Corinna Höfling, Markus Morawski, Ulrike Zeitschel, Elisa R. Zanier, Katrin Moschke, Alperen Serdaroglu, Fabio Canneva, Stephan von Hörsten, Maria‐Grazia De Simoni, Gianluigi Forloni, Carsten Jäger, Elisabeth Kremmer, Steffen Roßner, Stefan F. Lichtenthaler, Peer‐Hendrik Kuhn

**Affiliations:** ^1^ Paul Flechsig Institute for Brain ResearchUniversity of LeipzigLeipzigGermany; ^2^ Department of NeuroscienceIRCCSIstituto di Ricerche Farmacologiche Mario NegriMilanoItaly; ^3^ German Center for Neurodegenerative Diseases (DZNE)MunichGermany; ^4^ Institute for Advanced StudyTechnische Universität MünchenGarchingGermany; ^5^ Institut für Pathologie und Pathologische AnatomieTechnische Universität MünchenMunichGermany; ^6^ Department of Experimental TherapyPräklinisches Experimentelles Tierzentrum (PETZ)Universitätsklinikum ErlangenErlangenGermany; ^7^ Helmholtz Zentrum MünchenGerman Research Center for Environmental HealthInstitute of Molecular ImmunologyMunichGermany; ^8^ Neuroproteomics, Klinikum rechts der IsarTechnische Universität MünchenMunichGermany; ^9^ Munich Cluster for Systems Neurology (SyNergy)MunichGermany

**Keywords:** amyloid precursor protein, monoclonal antibody, immunohistochemistry, transgenic animal models, neuropathology, Alzheimer's disease

## Abstract

Alzheimer's disease (AD) is histopathologically characterized by neurodegeneration, the formation of intracellular neurofibrillary tangles and extracellular Aβ deposits that derive from proteolytic processing of the amyloid precursor protein (APP). As rodents do not normally develop Aβ pathology, various transgenic animal models of AD were designed to overexpress human APP with mutations favouring its amyloidogenic processing. However, these mouse models display tremendous differences in the spatial and temporal appearance of Aβ deposits, synaptic dysfunction, neurodegeneration and the manifestation of learning deficits which may be caused by age‐related and brain region‐specific differences in APP transgene levels. Consequentially, a comparative temporal and regional analysis of the pathological effects of Aβ in mouse brains is difficult complicating the validation of therapeutic AD treatment strategies in different mouse models. To date, no antibodies are available that properly discriminate endogenous rodent and transgenic human APP in brains of APP‐transgenic animals. Here, we developed and characterized rat monoclonal antibodies by immunohistochemistry and Western blot that detect human but not murine APP in brains of three APP‐transgenic mouse and one APP‐transgenic rat model. We observed remarkable differences in expression levels and brain region‐specific expression of human APP among the investigated transgenic mouse lines. This may explain the differences between APP‐transgenic models mentioned above. Furthermore, we provide compelling evidence that our new antibodies specifically detect endogenous human APP in immunocytochemistry, FACS and immunoprecipitation. Hence, we propose these antibodies as standard tool for monitoring expression of endogenous or transfected APP in human cells and APP expression in transgenic animals.

## Introduction

Alzheimer's disease (AD) is the most frequent neurodegenerative disorder worldwide. It is marked by the generation and deposition of small, neurotoxic Aβ peptides in form of oligomeric aggregates and finally plaques in the brain parenchyma and vasculature. The Aβ peptide results from the sequential proteolytic processing of the amyloid precursor protein (APP) by β‐ and γ‐secretases (Lichtenthaler *et al*., 2011; Vassar *et al*., 2014).

To understand mechanisms of Aβ generation and the effects of Aβ aggregates on neuronal function, animal models which mirror this aspect of AD are required. Such models are also of great interest for pharmacological studies that aim at reducing Aβ generation or even removing existing Aβ aggregates from the brain. However, when using rodent animal models, it has to be taken into account that Aβ from mice and rats contains three amino acid substitutions as compared to human Aβ (R5G, Y10F and H13R). These alterations were shown to influence APP processing (De Strooper *et al*., 1995; Reaume *et al*., 1996) and the ability of Aβ peptides to form secondary structures such as oligomers and fibrils (Dyrks *et al*., 1993; Otvos *et al*., 1993). This could explain the virtual absence of Aβ deposits in normal young and aged rodent brains. Therefore, transgenic models of Aβ pathology have been developed that overexpress human APP (hAPP) with AD‐associated mutations which favour the amyloidogenic β‐secretase pathway of APP processing (Games *et al*., 1995; Hsiao *et al*., 1996; Sturchler‐Pierrat *et al*., 1997). These mice have been used to test therapeutic strategies that aim at reducing Aβ generation, for example by treatment with β‐secretase inhibitors and by active and passive immunization approaches (Solomon *et al*., 1996; Schenk *et al*., 1999; Eketjall *et al*., 2013). Interestingly, transgenic mice overexpressing hAPP without AD‐related mutations, like I5, barely develop any Aβ deposits at high age (Mucke *et al*., 2000). However, mice overexpressing mutant hAPP Swedish display Aβ deposits starting at around 10 months of age, for example Tg2576 (Hsiao *et al*., 1996), while mice overexpressing mutant hAPP Swedish in combination with mutant γ ‐secretase components generate Aβ deposits even faster, for example 5xFAD (Oakley *et al*., 2006).

Although these models support the amyloid cascade hypothesis, clear differences can be observed among the mouse models with respect to the spatial and temporal appearance of Aβ deposits, synaptic dysfunction and impairments in memory and behaviour. We hypothesize that these differences are directly linked to the spatial and temporal expression pattern of hAPP and to its expression levels. This has recently been exemplified in a study that attributed APP transgene expression instead of BACE1 heterozygosity for lowered Aβ levels in APP‐transgenic mice crossed with BACE1 knockout mice (Sadleir *et al*., 2015). Until now no antibodies have been available which could convincingly discriminate ectopically expressed human from endogenous murine APP in immunocytochemistry and immunohistochemistry to detect these differences. In a study that compared several APP and Aβ antibodies (4G8, 22C11, Y188 and others) for their specificity in immunocytochemistry between wild‐type and APP knockout neurons, only the antibody Y188 specifically recognized APP (Guo *et al*., 2012). However, this antibody detects both overexpressed human and endogenous murine APP, making it impossible to distinguish among APP of different species. Hence, in this study we developed two rat monoclonal antibodies that recognize hAPP but not murine APP in Western blot, immunocytochemistry, immunohistochemistry, immunoprecipitation and FACS analyses under overexpression and, even more important, endogenous conditions. Applying these antibodies to tissue specimens of APP‐transgenic mouse and rat models, we unambiguously identify distinct neuronal subsets expressing the hAPP transgene in specific animal models. This may explain differences in Aβ pathology, synaptic dysfunction and learning and memory as well as behavioural deficits. In summary, we expect these hAPP‐specific antibodies to become important and versatile research tools for future AD animal model studies and studies in induced pluripotent stem cell‐derived neurons.

## Results

### Generation and characterization of hAPP‐specific rat antibodies

**Figure 1:**
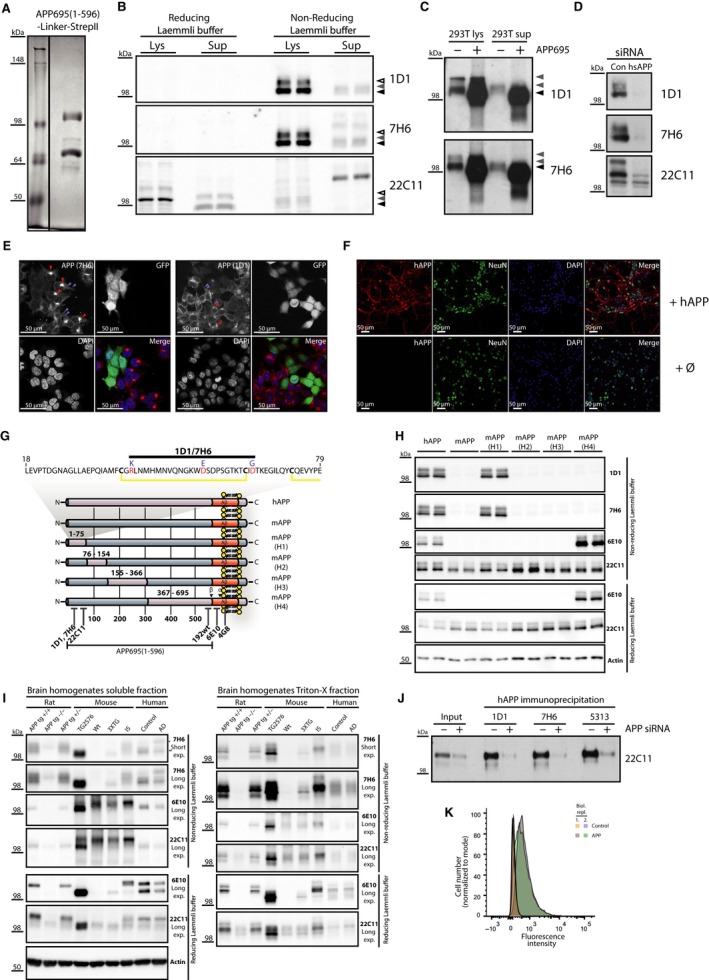
Validation of novel APP‐specific antibodies in different applications. (A) Quality control of hAPP‐linker‐Precission‐StrepII antigen on a Coomassie gel prior to vaccination (Left lane: molecular weight marker, right lane: hAPP‐linker‐Precission‐StrepII). (B) Validation of novel antibodies 1D1 and 7H6 for the detection of endogenous hAPP in HEK293T lysates (Lys) and conditioned supernatant (Sup) under reducing (reducing Laemmli buffer) and nonreducing (nonreducing Laemmli buffer) conditions by Western blot analysis. 1D1 and 7H6 detected a specific band for APP in conditioned media (grey arrowhead) and two bands immature (black arrowhead) and mature APP (empty arrowhead) in cell lysates of HEK293T (293T) cells all above 98 kDa only under nonreducing conditions while 22C11 detected a specific signal in lysates and conditioned media only under reducing conditions. (C) Specificity of the novel antibodies was further validated comparing HEK293T cells (−) with HEK293T cells overexpressing hAPP695 (+). Both antibodies detected endogenous APP751 and APP770 in HEK293T (grey arrowheads) and overexpressed APP695 at a slightly lower molecular weight as a strong increase of the 98 kD bands. Sup: supernatant. (D) Specificity of both antibodies was tested in HEK293T cell lysates with a siRNA‐mediated APP knockdown. APP knockdown was validated with 22C11 which shows additional remaining unspecific bands. 1D1 and 7H6 bands were completely abolished upon APP knockdown. (E) Specificity of both antibodies was tested in immunocytochemistry. HEK293T cells with a lentivirus‐mediated APP knockdown and GFP expression were mixed with wild‐type HEK293T cells and stained with 7H6 and 1D1. Both antibodies show a Golgi (red arrows) and vesicular staining (light blue arrows) which is abolished upon APP knockdown (see GFP‐positive cells; APP red, DAPI blue). (F) Both antibodies were tested for their specificity towards hAPP in primary cortical neurons of mice. Only upon lentiviral overexpression of hAPP, a specific signal could be observed with the antibody 7H6, while in the nontransduced control neurons (Ø), no signal was detected. 1D1 showed the same staining pattern. (G) We identified the epitope of 7H6 and 1D1 creating chimeric APP constructs by the exchange of distinct parts of murine APP for human APP (mAPP‐(H1‐H4)). These constructs were expressed together with murine and hAPP in HEK293T cells. Pink colour indicates hAPP sequence; blue colour indicates murine APP sequence. (H) While 22C11 detected all constructs and 6E10 detected hAPP and mAPP‐H4 under reducing and nonreducing conditions, 7H6 and 1D1 detected only hAPP and the chimeric APP construct mAPP‐H1 under nonreducing conditions which shows that the epitope lies between amino acid 1–75. The proposed binding epitope is depicted above the chimeric APP constructs. (I) 1D1 and 7H6 were tested and compared to 22C11 and 6E10 for their specificity towards hAPP in brain homogenates of wild‐type mice, APP‐transgenic mouse models, an APP‐transgenic rat model and human healthy and AD brains under reducing and nonreducing conditions by Western blot analysis. 22C11 detected a signal in all mice and rats as well as human brains properly only under reducing conditions but not under nonreducing conditions due to the presence of additional background bands. 6E10 properly detected APP only in transgenic animals under reducing conditions but detected additional strong unspecific bands under nonreducing conditions in mouse models which made discrimination between wild‐type and APP‐transgenic mice difficult except for Tg2576 mice which heavily overexpress hAPP. 7H6 only detected a clear signal in transgenic mice and rats. Furthermore, 7H6 detected a shift of APP695 (*) towards the APP770 isoform (**) between healthy and AD human brains which reflects neuronal loss and astrogliosis in AD pathogenesis. (J) Antibodies 7H6 and 1D1 were tested and compared to the polyclonal antibody 5313 for their ability to immunoprecipitate hAPP from conditioned media of HEK293T cells. About 20 μL of directly loaded supernatants of HEK293T cells (input) transfected with a control (−) or an APP‐specific siRNA (+) was compared to immunoprecipitated hAPP of 200 μL medium for each antibody. Specificity of immunoprecipitated material was proven by the hAPP‐specific siRNA‐mediated knockdown. (K) 7H6 antibody was tested for its applicability in FACS. Overexpression of hAPP led to a clearly detectable increase of the 555 signal (shift towards the right). Orange and light blue indicate biological replicates of control cells, and grey and green indicate biological replicates of APP overexpressing cells.

For hAPP‐specific antibody generation, the ectodomain of the neuronal isoform hAPP695 lacking the KPI domain was fused with a StrepII tag and subsequently stably expressed in HEK293T cells. We were able to purify 1.5 mg of pure hAPP ectodomain of which 1 μg was analysed with a Coomassie gel as a quality control (Fig. 1A). The intact hAPP ectodomain with an apparent molecular weight of 100 kDa and additional smaller degradation products in the molecular weight range of 80–60 kDa were detected. The purified hAPP ectodomain was injected into male Lou rats twice. After immunization, 10 hybridoma clones were obtained whose supernatants detected recombinant native hAPP in ELISA. All of these clones worked as well under nonreducing conditions in Western blot analyses (not shown).

The two most promising monoclonal antibodies (1D1 and 7H6) were rigorously tested in various applications. First, endogenous hAPP was detected in cell lysates and supernatants of HEK293T cells by Western blot analysis. Similar to the hAPP ectodomain‐binding antibody 22C11, both novel rat antibodies 7H6 and 1D1 detected endogenous hAPP. However, in contrast to 22C11, 7H6 and 1D1 work only under nonreducing conditions which suggests a conformational epitope being recognized by both antibodies (Fig. 1B). Next, the specificity of both antibodies was tested in HEK293T cells overexpressing hAPP695. Both antibodies were able to detect hAPP overexpression in cell lysates and supernatants. Thus, 1D1 and 7H6 detect endogenous and overexpressed membrane bound and soluble hAPP on Western blots. Ectopically expressed APP695 lacking the KPI domain (black arrow) had a slightly smaller molecular weight than endogenous APP751 and APP770 (grey arrow) (Fig. 1C). Finally, the specificity of both antibodies was proven by Western blot analysis after knocking down endogenous hAPP with an APP‐specific siRNA (Fig. 1D). The knockdown efficiency was first tested with a 22C11 blot. hAPP‐specific bands disappeared while unspecific bands remained. In contrast to 22C11, both 1D1 and 7H6 gave only two bands in Western blots which represent mature and immature APP and both disappeared under hAPP knockdown conditions (Fig. 1D).

To our knowledge, there is no APP ectodomain targeting monoclonal antibody available which recognizes endogenous hAPP specifically in immunocytochemistry. However, a C‐terminal monoclonal rabbit antibody has been developed (Y188, Epitomics) and is able to specifically recognize human and murine APP in immunocytochemistry and immunohistochemistry (Guo *et al*., 2012). We tested both novel rat antibodies for their ability to detect endogenous hAPP in HEK293T cells (Fig. 1E). Therefore, we created stable APP knockdown HEK293T cells via lentiviral transduction. The lentiviral vector additionally expressed GFP, which allowed to track transduction efficiency and thus to indirectly monitor APP knock‐down due to shRNA expression. Transduced APP knockdown cells and nontransduced HEK293T cells were mixed in a 1:2 ratio and plated in 96‐well plates for testing the specificity of both antibodies in immunocytochemistry. Confocal images revealed staining within the Golgi (red arrows) as well as intracellular vesicles (light blue arrow) (Fig. 1E, APP channel). APP staining was only observed in nontreated HEK293T cells, while the hAPP‐specific signal was almost completely abolished in GFP‐positive hAPP knockdown cells (Fig. 1E). Furthermore, primary cortical neurons overexpressing hAPP695 were labelled with 7H6 antibody after transduction of hAPP695 with the Gal4‐UAS lentiviral system. While wild‐type neurons showed no staining with the antibody 7H6 or 1D1, hAPP‐overexpressing neurons displayed a distinct and intense staining for hAPP (Fig. 1F) which indicated that both antibodies recognize human but not murine APP.

We were interested in understanding how 7H6 and 1D1 compared to well‐established antibodies like 22C11 and 6E10 were able to discriminate between human and murine APP on the molecular level. To this aim, we compared the amino acid sequence of hAPP to the amino acid sequence of APP in several other species *in silico*. Based on this analysis, several APP chimeras [mAPP(H1)‐(H4)] were created swapping those regions of the murine APP sequence for hAPP that contained specific amino acid exchanges between the two species (indicated in the diagram showing the APP membrane topology including the humanized domains) (Fig. 1G). Western blot analysis of HEK293T cells overexpressing hAPP, murine APP (mAPP) and the APP chimeras mAPP(H1)‐(H4) revealed that only overexpressed hAPP and the APP chimera mAPP(H1) were detected both by 7H6 and 1D1 while 22C11 detected all constructs and 6E10 detected hAPP and the APP chimera mAPP(H4) both under reducing and nonreducing conditions (Fig. 1H). This indicates that the epitope for both antibodies is located between amino acid positions 1–75 of hAPP. As mature APP lacks the signal peptide (1–17), the epitope can be narrowed down to amino acid position 18–75. Furthermore, we observed that both novel antibodies work only under non‐reducing conditions in Western blot which indicates that a disulphide bridge plays a decisive role for the recognized epitope of both antibodies. Within the region 1–75, cysteines 38 and 62 form a disulphide bridge. Going back to the *in silico* alignment of the APP amino acid sequence between human and other species, we found three amino acids (K40, D53, D64) that are specific for hAPP (red) and whose variants (R40, E53, G64) (blue) are conserved among other species. Thus, we are convinced that the epitope must lie in between amino acids 40 and 64 (Fig. 1G).

Next, 7H6, 6E10 and 22C11 were tested on brain homogenates of wild‐type and frequently used hAPP‐transgenic mouse and rat models (Hsiao *et al*., 1996; Mucke *et al*., 2000; Oddo *et al*., 2003; Leon *et al*., 2010) under reducing and nonreducing conditions. Under nonreducing conditions 7H6 detected an APP‐specific signal only in transgenic mouse models, a transgenic rat model and human brain tissue both in the PBS soluble and the triton‐soluble fraction while 6E10 and 22C11 besides APP both detected additional nonspecific bands in mouse brains irrespective of the genotype which made a clear distinction of the genotype especially between wild‐type and 3XTG mice almost impossible (Fig. 1I). However, in rat brains 6E10 was able to clearly distinguish between transgenic and nontransgenic brain homogenates due to a lack of nonspecific bands. Additionally, 7H6 detected a decrease of neuronal APP695 (**) and a concomitant increase of glial APP751 (*) in the PBS soluble fraction of human AD brains compared to healthy human brain specimens which we did not observe with any of the other antibodies (Fig. 1I). This finding very likely results from neurodegeneration in combination with a reactive gliosis in the course of AD. In contrast to nonreducing conditions, 6E10 demonstrated its well‐known specificity for hAPP under reducing conditions by clearly distinguishing between transgenic and nontransgenic mouse and rat models. Both antibodies were additionally tested in further applications. For testing their use in immunoprecipitation, endogenous hAPP was precipitated from conditioned supernatants of HEK293T cells transfected with a hAPP‐specific siRNA or no siRNA (Fig. 1J). As a positive control, a rabbit polyclonal serum 5313 was included which has been described previously (Kaether *et al*., 2002). In control siRNA‐transfected HEK293T cells, both monoclonal rat antibodies 1D1 and 7H6 precipitated the hAPP ectodomain with a similar efficiency as the polyclonal 5313 serum. The specificity of the immunoprecipitated product was proven by its clear reduction in the hAPP knockdown cells (Fig. 1J).

7H6 was also tested for its application in fluorescence‐activated cell sorting (FACS). When comparing the staining of HEK293T cells and HEK293T cells overexpressing hAPP695, a clear difference was observed demonstrating the applicability of 7H6 for FACS (Fig. 1K).

### Application of rat anti‐hAPP antibodies to monitor transgene expression in transgenic animal models

Both novel antibodies were also examined for their applicability in immunohistochemistry to monitor the spatial expression pattern of the hAPP transgene in different mouse and rat models. Therefore, both antibodies were compared to the established APP antibody 22C11 on brain sections of wild‐type, I5, Tg2576 and 3xTg mice. Additionally, the antibody 6E10 was included in our comparative analysis as 6E10 is frequently considered to be specific for hAPP. We first tried to identify the optimal 6E10 antibody concentration for distinguishing properly between wild‐type and APP‐transgenic animals. Serial antibody dilutions of 6E10 (2–0.2 μg mL^−1^) and of 1D1 (30–3.75 μg mL^−1^) were used on brain tissue of wild‐type and Tg2576 APP‐transgenic mice which have the highest APP expression among all investigated mouse models in this study (Fig. S1). 1D1 staining did not result in unspecific labelling in wild‐type mice but showed robust staining of neurons in Tg2576 mice at all investigated dilutions. In contrast, 6E10 produced strong nonspecific background staining at low dilutions and a neuronal staining at higher dilutions in wild‐type mice and labelling of neurons at all concentrations in transgenic mice with a decrease in background staining as 6E10 was further diluted. Thus, 6E10 staining did not allow a clear distinction between Tg2576 APP‐transgenic and wild‐type mice at any of the tested concentrations. We, therefore, further compared 1D1 to the 22C11 antibody which binds close to the APP N‐terminus and thus is more suitable to benchmark our newly developed APP N‐terminus‐specific antibodies 1D1 and 7H6. As 1D1 and 7H6 generated identical staining patterns in all brain tissues, only 1D1 immunolabelling is presented in the figures.

**Figure 2:**
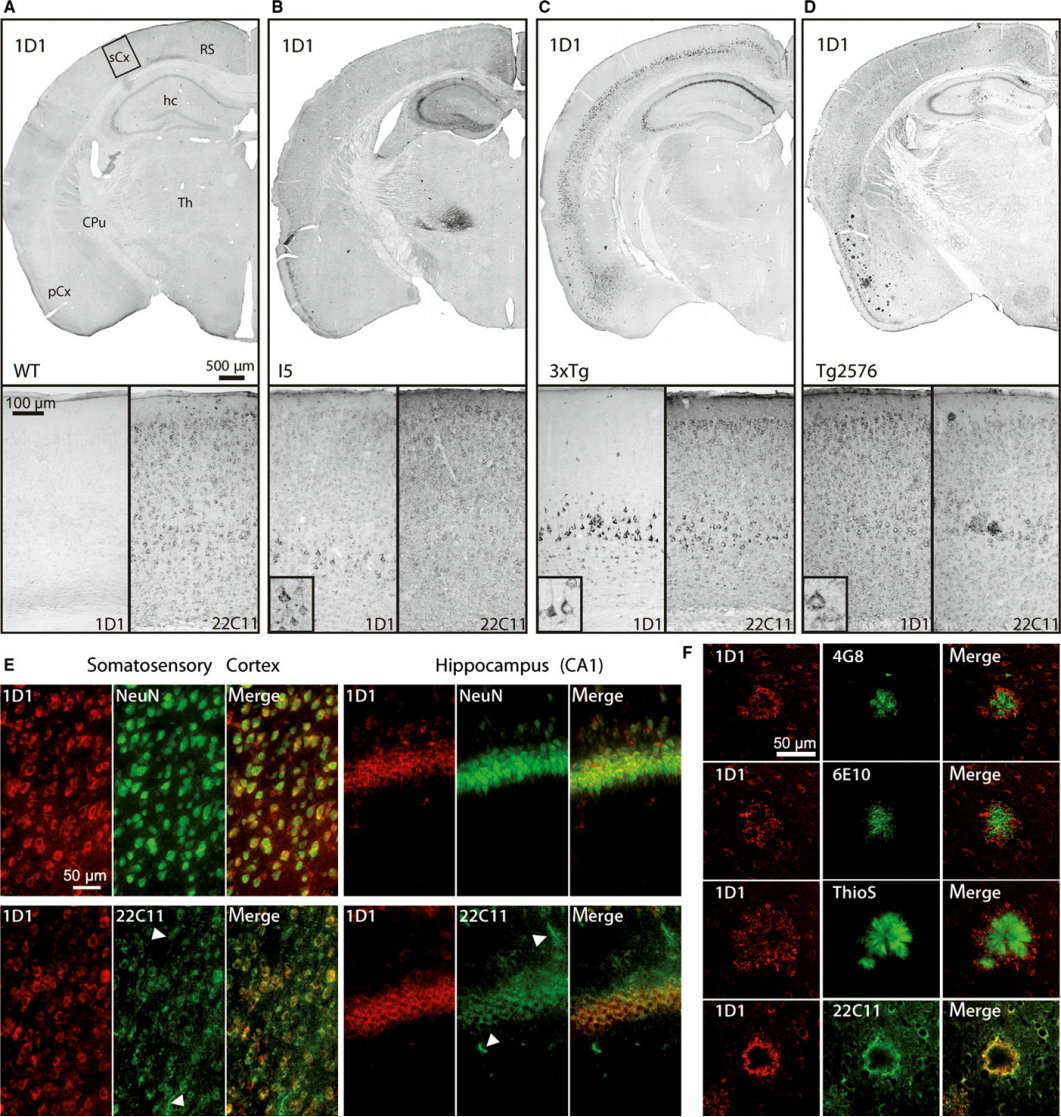
Immunohistochemistry reveals mouse line‐specific hAPP expression. (A) In brains of wild‐type (WT) mice, the hAPP‐specific antibody 1D1 does not label any structures, whereas 22C11 labels numerous neurons. (B) In the brain of I5 mice, numerous neocortical neurons are labelled by the hAPP‐specific antibody 1D1. The transgene is specifically expressed by many neocortical neurons, hippocampal CA2/3 and dentate gyrus neurons as well as neurons in thalamus. (C) In 3xTg mice, the hAPP expression in neocortex is restricted to layer V pyramidal neurons and in hippocampus to CA1 and to a lesser extent CA2/3 neurons. (D) In Tg2576 mice, hAPP is expressed by neurons across all neocortical layers and in hippocampal CA1 to 3 regions. Additionally, 1D1 labels numerous plaques in the piriform cortex. (E) *Top*: In Tg2576 brain, double immunofluorescent labelling of hAPP by 1D1 and neurons by NeuN reveals the neuron‐specific expression of hAPP in somatosensory cortex (left) and hippocampus (right). *Bottom*: In Tg2576 mice, double immunofluorescent labelling of hAPP by 1D1 and mouse/human APP/APLP‐2 by 22C11 demonstrates the labelling of non‐neuronal structures by 22C11 (arrowheads) but not by 1D1 in somatosensory cortex (left) and hippocampus (right). The scale bars in the images apply to all corresponding microphotographs. The scale bar in the inset in (B) represents 20 μm. (F) Double labelling of hAPP by 1D1 (red) in combination with the Aβ‐specific antibodies 4G8 and 6E10, with thioflavin S (ThioS) and with the N‐terminal antibody 22C11 (all in green). Note the labelling of the plaque core by 6E10, 4G8 and ThioS and the labelling of dystrophic neurites in the plaque periphery by 1D1. The 1D1 antibody generates an identical labelling as 22C11. The scale bar represents 50 μm and applies to all images. sCx somatosensory cortex; RS retrosplenial cortex; hc hippocampus; CPu caudate putamen; Th thalamus, pCx piriform cortex

While immunohistochemical labelling with 22C11 in neocortex of I5 and Tg2576 mice did not differ significantly from that in wild‐type mice (Fig. 2A,B,D), in 3xTg mice only the robust 22C11 labelling of layer V pyramidal neurons differed from those in wild‐type brains (Fig. 2C). This antibody additionally detected dystrophic neurites around plaques in brain sections of Tg2576 mice but not in I5 mice that are devoid of Aβ deposits or of 3xTg mice which only develop Aβ deposits at high age.

In contrast to 22C11, 1D1 as mentioned above generated no labelling in brain specimens of wild‐type mice (Fig. 2A, Fig. S1), but prominent and distinct neuronal labelling in all three APP‐transgenic mouse lines analysed (Fig. 2B–D). For example, 1D1 labelled numerous neurons in the neocortex and piriform cortex of I5 and Tg2576 mice, but only pyramidal layer V neurons in 3xTg mice. This differential transgene expression could not be demonstrated using the antibody 22C11. Moreover, in the hippocampal formation, transgenic hAPP expression was most prominent in CA2/3 and dentate gyrus granule neurons of I5 mice, in CA1 to CA3 pyramidal neurons of Tg2576 mice and in CA1 neurons and to a lesser extent CA2/3 neurons in 3xTg mice. In addition, I5 mice display hAPP immunoreactivity in the thalamus which was not detected in Tg2576 and 3xTg mice.

Transgenic hAPP was specifically detected in neurons as shown by co‐localization with the neuronal marker NeuN in cortex and hippocampus (Fig. 2E) which is in line with neuron‐specific promoter driven transgene expression and provides further evidence for the specificity of the 1D1 antibody. Additionally, 1D1 generated a more distinct membranous labelling of cortical and hippocampal neurons than 22C11 and did not label glia‐like structures marked by 22C11 (Fig. 2E). To analyse the structures labelled by 1D1 in association with Aβ plaques, double labellings of the 1D1 antibody with Aβ antibodies 6E10 and 4G8, with the fibril‐labelling dye thioflavin S and with the N‐terminal APP antibody 22C11 were performed in Tg2576 mice. While the Aβ antibodies and thioflavin S labelled the core of plaques, the 1D1 antibody generated a more peripheral, diffuse extracellular plaque staining and a distinct labelling resembling most likely Aβ plaque‐associated dystrophic neurites (Fig. 2F). Interestingly, both 1D1 and 22C11 antibodies displayed an identical staining pattern, consistent with the labelling of APP, but not of Aβ by these antibodies (Fig. 2F). This is in agreement with the presence of APP in subsets of plaques and plaque‐associated dystrophic neurites as reported earlier (Joachim *et al*., 1991; Rozemuller *et al*., 1993).

The 1D1 antibody was further used to investigate transgenic hAPP expression during aging of Tg2576 mice between postnatal month 3 and 20 by Western blot analysis and immunohistochemistry. For Western blot analyses, left brain hemispheres without cerebellum were used to prepare homogenates. The quantification of Western blots revealed a slight, statistically not significant reduction of hAPP content between postnatal month 11 and 20, compared to younger mice (Fig. S2). Immunohistochemistry demonstrated a similar cell type‐specific staining pattern in Tg2576 brain across all ages and the labelling of dystrophic neurites from 16 months onwards (Fig. S2).

To reveal a potential modulation of transgenic hAPP expression after experimental manipulation, a model of controlled cortical impact brain injury was assessed. We observed the induction of hAPP transgene expression in neurons at the site of injury that allowed monitoring changes in neuronal morphology (Fig. S3).

In transgenic mouse models overexpressing mouse APP with the murine or humanized Aβ sequence (Xu *et al*., 2015), no labelling of neurons or of Aβ plaque‐associated structures was detected using 1D1 or 7H6 antibodies (not shown).

**Figure 3:**
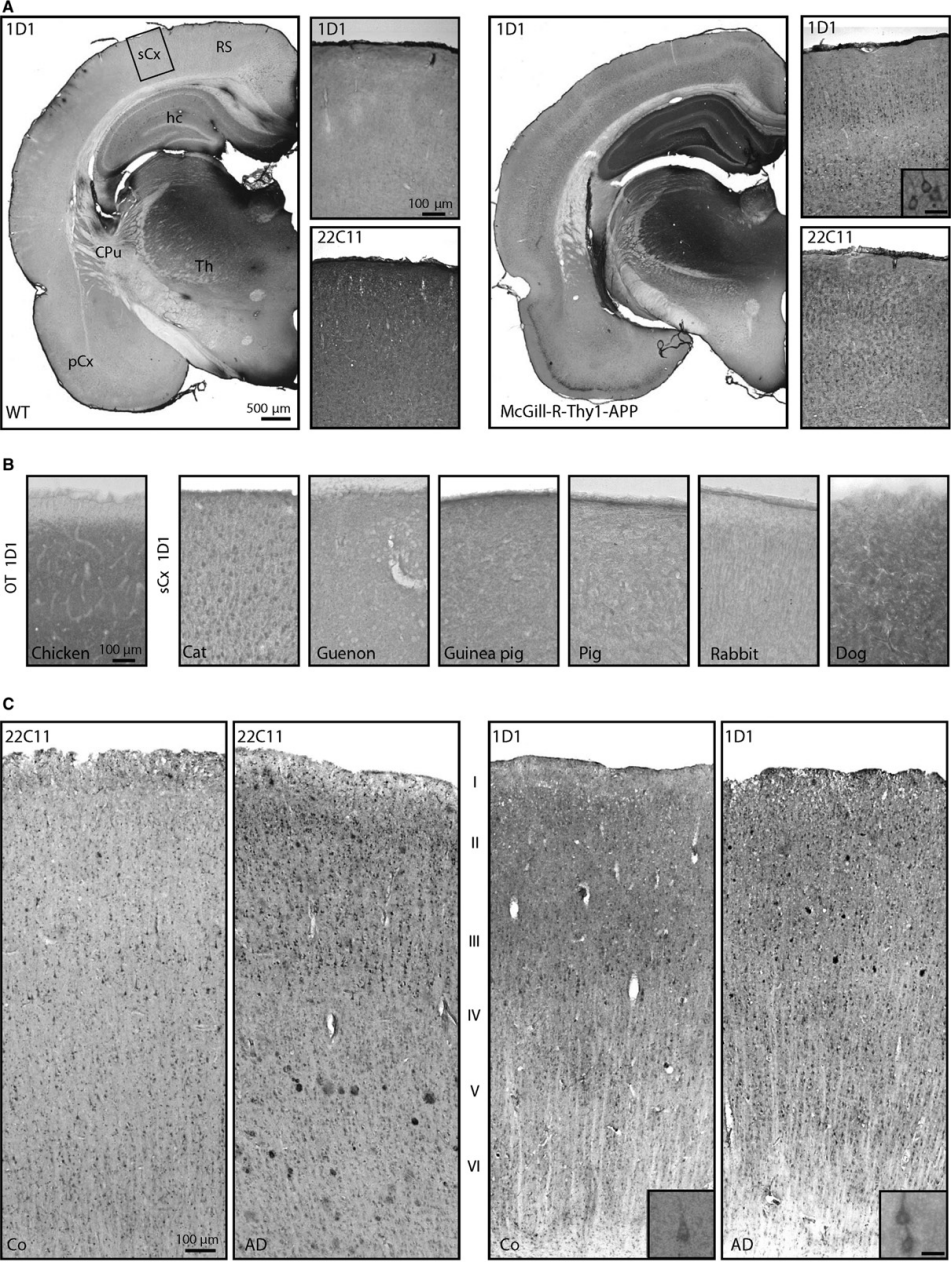
Immunohistochemistry for hAPP in hAPP‐transgenic rats, nontransgenic animal species and human control and AD brain. (A) In the brain of McGill‐R‐Thy1‐APP rats (right), numerous neocortical neurons are labelled by the hAPP‐specific antibody 1D1. Diffuse hAPP immunoreactivity is also present in hippocampus. This labelling was not detected in brains of wild‐type (WT) littermates (left). (B) In somatosensory cortex of different nontransgenic animal species, 1D1 only generated specific signals in cat, but not in chicken, guenon, guinea pig, pig, rabbit and dog. (C) In human neocortical brain tissue from control (Co) and AD subjects, 22C11 (left) and 1D1 (right) generated similar staining patterns for neurons. Both antibodies also labelled Aβ plaques. The scale bars in the images apply to all corresponding microphotographs. The scale bar in the inset in (A) represents 20 μm and also applies to the insets in (C). sCx somatosensory cortex; OT optic tectum; RS retrosplenial cortex; hc hippocampus; CPu caudate putamen; Th thalamus, pCx piriform cortex.

Another animal species used for transgenic hAPP expression is rat. Using the 1D1 antibody in McGill‐R‐Thy1‐APP rats, we demonstrate robust labelling in neocortex which was most prominent in layer V pyramidal neurons and weakest in layer IV (Fig. 3A). In hippocampus, strong but diffuse immunoreactivity was present, which may be indicative of substantial APP ectodomain shedding and release into the extracellular space. In brains of wild‐type littermates, only weak background staining and occasional labelling of blood vessels was detected by the 1D1 antibody rat (Fig. 3A). This further demonstrates the specificity for human – but not mouse and rat – APP and underlines the usefulness of 1D1 and 7H6 antibodies for mapping APP transgene expression in these animal species.

There is growing attention to nontransgenic animals with a human Aβ peptide sequence as more physiological models for sporadic AD (Beck *et al*., 2003; Sarasa & Pesini, 2009; Moreno‐Gonzalez & Soto, 2012; Sharman *et al*., 2013; Bates *et al*., 2014). To reveal a possible cross‐reactivity of 1D1 with APP of other animal species, brain sections of chicken, cat, guenon, guinea pig, pig, rabbit and dog were analysed by immunohistochemistry. As shown in Fig. 3B, only in the somatosensory cortex of cat a labelling was generated by the 1D1 antibody indicating a rather hAPP‐specific epitope for the antibody.

In human brain tissue of control and AD cases, strong labelling of neocortical neurons was observed using both 22C11 and 1D1 antibodies (Fig. 3C). There were no obvious differences in the structures labelled or in staining intensity between 22C11 and 1D1. In AD brain, both 22C11 and 1D1 also labelled plaque‐associated dystrophic neurites as already described above for Tg2576 mice.

## Discussion

Within this study, we have developed two novel rat monoclonal antibodies (7H6 and 1D1) raised against the native hAPP ectodomain which both recognize human but not APP of other species such as mouse, rat, dog or guinea pig. Both antibodies have been rigorously tested in Western blot, immunoprecipitation, FACS, immunocytochemistry, immunohistochemistry and ELISA. Therefore, these antibodies are an ideal tool to study the hAPP protein in many *in vitro* and *in vivo* models.

We have shown that 7H6 and 1D1 detect endogenous APP in immunocytochemistry by discriminating APP knockdown from wild‐type HEK293T cells based on the APP signal in a mixture of wild‐type and APP knockdown HEK293T cells. Hence, we are convinced that both antibodies can be used to study trafficking, function and expression of the hAPP protein in patient fibroblasts and induced pluripotent stem cells (iPS) derived thereof as well as in neurons of AD patients and healthy individuals to study the behaviour or localization of normal or mutant hAPP via microscopy (Israel *et al*., 2012; Shi *et al*., 2012; Kondo *et al*., 2013). Currently, to the best of our knowledge, there is only one commercial APP antibody called Y188 directed towards an epitope of the APP cytoplasmic domain which has been shown to be specific for APP in immunocytochemistry while many other tested antibodies like 22C11 are not (Guo *et al*., 2012). However, Y188 cannot discriminate human and murine APP and binds to the intracellular domain which prohibits staining of vital cells. Moreover, this feature of Y188 precludes the detection of soluble N‐terminal hAPP fragments secreted from cells, whereas 1D1 and 7H6 bind to the N‐terminus of APP and, thus, detect soluble full length and secreted hAPP. Additionally, as we were able to distinguish ectopically expressed hAPP from endogenous murine APP in primary cortical neurons, both antibodies might be used to study the precise distribution of transgenic APP within the synapse on the molecular level via super resolution microscopy or electron microscopy *in vitro* (Tonnesen & Nagerl, 2013). Furthermore, we demonstrate that both antibodies work in immunoprecipitation. Hence, these antibodies could be used to isolate APP cleavage products of ectopically expressed hAPP out of APP‐transgenic brain homogenates or to identify potential binding partners of APP.

Furthermore, we clearly demonstrate that both 1D1 and 7H6 in comparison with 22C11 and 6E10 are able to specifically recognize transgenic APP expression in APP‐transgenic animal models. 6E10 has been proposed to be specific for hAPP. Indeed, in reducing Western blot this is the case (Fig. 1I). However, 6E10 detects background bands under nonreducing conditions which prevent a clear discrimination between wild‐type and transgenic mice except for Tg2576 mice. Nonreducing conditions in Western blots resemble the conditions during immunohistochemistry and immunocytochemistry more closely and thus are more indicative for the performance of antibodies in these applications. In contrast to reducing conditions in Western blot, we obtained unspecific background bands under nonreducing conditions with 6E10. Nevertheless, we tried to optimize detection conditions of 6E10 by testing different concentrations. However, in contrast to our new antibody 1D1 none of the tested concentrations produced a satisfying result in our hands (Fig. S1). We thus conclude that 6E10 is suitable to discriminate human and mouse APP only under reducing conditions in Western blot analysis but not in immunohistochemistry and immunocytochemistry.

APP‐transgenic animal models for AD have been available for 20 years and provided the basis for a better understanding of APP processing and the effects of different types of Aβ aggregates on synaptic dysfunction, learning and memory (Weidemann *et al*., 1989). One of the first AD mouse models introduced solely overexpresses the neuronal isoform hAPP695, which additionally carries the Swedish mutation at the β‐cleavage site (Hsiao *et al*., 1996). Other models ectopically express the hAPP695 isoform combining the Swedish and the Indiana mutation in the Aβ domain (Hsia *et al*., 1999). However, late deposition of plaques in these models was the incentive to create mouse models that additionally carry familial AD‐related mutations in presenilins. These mutations modulate the Aβ40/Aβ42 ratio towards Aβ42 and thus accelerate Aβ aggregate formation. However, besides Aβ pathology, intraneuronal aggregates of hyperphosphorylated microtubule‐associated protein tau, so‐called neurofibrillary tangles, are another histopathological feature of AD. Hence, a triple transgenic model that overexpresses the aforementioned hAPP Swedish and AD‐specific mutants of presenilin and tau has been created (Oddo *et al*., 2003). Other mouse models that express hAPP without AD‐specific mutations and do not develop Aβ plaques were created to differentiate between pathological effects of hAPP overexpression and Aβ aggregate formation (Mucke *et al*., 2000). Although these AD mouse models are based on the amyloid cascade hypothesis and on similar AD‐related mutations, the phenotypic outcome is different.

We propose that these differences partially result from the expression level and the spatiotemporal expression pattern of the hAPP transgene. However, antibodies frequently used for the immunohistochemical labelling of APP additionally recognize Aβ peptides (such as 4G8 and 6E10) or the amyloid precursor like protein‐2 (such as 22C11) (Slunt *et al*., 1994; Webster *et al*., 1995) (Fig. 1G). In contrast, both 1D1 and 7H6 exclusively bind to amino acid 40–64 of hAPP in its native state and thus do not detect Aβ. This favourable feature of these novel antibodies precludes the detection of transgene expression and Aβ deposition in mouse models expressing mouse APP with a humanized or murine Aβ sequence (Xu *et al*., 2015).

The variable impact of hAPP transgene expression on memory performance, behaviour and cognition in different APP‐transgenic mouse models speaks in favour of differences in spatiotemporal expression of the hAPP transgene among these mouse models. Indeed, using our novel rat monoclonal antibodies 1D1 and 7H6, we could for the first time show that spatial hAPP transgene expression can vary a lot between different AD mouse models.

For example, 3xTg mice show intraneuronal Aβ deposition first in the neocortex at an age around 3–4 months. This coincides with cognitive impairment at 4 months as a deficit in long‐term retention and correlates with the accumulation of intraneuronal Aβ in the CA1 region of hippocampus and amygdala (Billings *et al*., 2005). At around 6 months, extracellular amyloid plaques become apparent in layers IV and V of the neocortex and later on as well in the CA1 region of the hippocampus of 3xTg mice which coincides with a decreased LTP and impaired synaptic transmission to age matched wild‐type littermates (Oddo *et al*., 2003; Billings *et al*., 2005). This spatiotemporal course of Aβ deposition correlates well with the transgene expression pattern of hAPP observed with the antibody 1D1 which strongly labelled pyramidal neurons in layer V and CA1 of the hippocampus, while CA2 and CA3 are almost devoid of APP transgene expressing neurons (Fig. 2). In contrast, 1D1 staining of brain specimens of Tg2576 mice revealed neuronal staining in the whole neocortex, hippocampus CA1‐CA3 and subcortical structures which correlates to the more evenly distributed deposition of amyloid plaques in Tg2576 mice (Kawarabayashi *et al*., 2001). This finding is in line with an earlier onset of LTP deficits in the dentate gyrus and dendritic spine loss in the CA1 region of the hippocampus (Jacobsen *et al*., 2006). Impaired spatial learning, working memory and contextual fear conditioning were observed at the same age (King & Arendash, 2002) which is well before extracellular plaques appear in the brain of these mice starting in limbic and cortical structures at the age of 10 months (Hsiao *et al*., 1996; Kawarabayashi *et al*., 2001). We were also able to detect APP transgene expression in I5 mice which do not develop plaques but still show synaptic deficits later in age (Mucke *et al*., 2000). In addition, the widespread hAPP expression in Tg2576 mice compared to I5 and 3xTg mice is reflected by the intense labelling on Western blots (Fig. 1I).

Many APP‐transgenic mouse models have been crossed to other transgenic/knockout mouse models to study their effect on amyloid deposition/clearance (Holtzman *et al*., 2012). However, in these studies, an effect on hAPP transgene expression could not be excluded as no antibody was available that enabled clear discrimination between human and murine APP. Both novel antibodies will be able to exclude effects on APP transgene expression and thus provide a valuable tool to control for effects that depend on modification of APP transgene expression rather than modification of APP and Aβ metabolism.

The novel hAPP‐specific antibodies also specifically recognize the hAPP transgene in a transgenic rat model. These rats display early intraneuronal Aβ accumulation in neocortex and hippocampus and extracellular Aβ deposits surrounded by activated glial cells and dystrophic neurites by 6 months of age (Leon *et al*., 2010). Interestingly, deficits in Morris water maze were already detected before the onset of Aβ plaque formation which is consistent with hAPP expression detected in hippocampus and prefrontal cortex and with oligomeric or fibrillar Aβ variants being synaptotoxic.

In summary, the novel antibodies presented here are suggested to be used as a standard tool to (i) reveal hAPP transgene expression patterns in existing and novel transgenic mouse and rat animal models, (ii) validate temporal expression transgene patterns during aging of transgenic animals and after breeding for multiple generations, (iii) compare hAPP transgene expression between different mouse lines, (iv) spatially relate physiological/pathological events, behavioural alterations in transgenic animals to transgene expression and (v) study the behaviour of APP regarding its trafficking, co‐localization with other molecules or mutations in patient iPS cell‐derived neurons or human cell lines.

## Experimental procedures

### Experimental animals

To reveal potential differences in transgene expression of hAPP‐transgenic mouse lines, we used 17‐ to 19‐month‐old mice driving transgene expression by the PDGF‐α chain promoter (I5), the hamster prion protein promoter (Tg2576) or the mouse Thy1.2 promoter (3xTg) (Table 1). These mice are all of C57BL/6 (or C57BL/6xSJL in case of Tg2576 and C57BL/6x129sv in case of 3xTg) background and express hAPP770 wild‐type (I5) or hAPP695 Swedish (Tg2576 and 3xTg). Additionally, a hAPP‐transgenic rat model was analysed at the age of 18 months. The McGill‐R‐Thy1‐APP rat expresses hAPP751 carrying the Swedish and Indiana mutations under control of the Thy1.2 promoter (Leon *et al*., 2010). In addition, neocortical brain sections of adult chicken, cat, guenon, guinea pig, pig, rabbit and dog were analysed by immunohistochemistry.

**Table 1:** Human APP‐transgenic mouse and rat models analysed

	Animal model					
	Wild‐type mouse	I5 mouse	Tg2576 mouse	3xTg mouse	Wild‐type rat	McGill‐APP rat
Promoter	–	PDGF α chain	Hamster prion protein	Mouse Thy1.2	–	Rat Thy1.2
Transgene	–	hAPP770wt	hAPP695swe	hAPP695swe	–	hAPP751swe/ind
Genetic background	C57BL/6	C57BL/6	C57BL/6 × SJL	C57BL/6 × 129sv	Wistar	Wistar

### Human brain tissue

Human brain tissue used for immunohistochemistry and Western blot analysis was obtained from the Banner Sun Health Research Institute Brain Donation Program (AD case: male, 77 years; control case: male, 82 years).

### Isolation of primary neurons

C57BL/6 wild‐type mice used for preparation of primary neurons were obtained from The Jackson Laboratory. All animal experiments were performed according to the European community council directive (86/609/ECC). Neurons were isolated as described previously (Mitterreiter *et al*., 2010) at E15/E16 and cultured in neurobasal medium supplemented with 2% B27, 100 U mL^−1^ penicillin, 100 μg mL^−1^ streptomycin and 0.5 mm glutamine. Experiments were carried out after 4–7 days *in vitro* (DIV).

### Lentivirus production

Viruses were produced by transient triple transfection of the lentiviral transfer vectors (FU‐APP, plKOmod2‐EGFP‐WPRE‐hsAPP‐2603, FhSyn‐Gal4‐VP16 or FKP/UAS‐APP) with psPAX2 and pcDNA3.1‐VSVG into HEK293T cells as previously described (Kuhn *et al*., 2010). For purification of viral stocks, typically eight 10‐cm dishes with 9 × 10^6^ HEK293T cells each were transiently transfected and the resulting virus was concentrated in 500 μL TBS.

### Transduction of primary cortical neurons

Primary neurons were co‐transduced after 2 DIV with purified virus stocks of FhSyn‐Gal4‐VP16 and FKP/UAS‐APP at a dilution of 1:200 or no virus. After transduction, neurons were cultured another 5 days until analysis.

All other experimental methods including hAPP antigen cloning and expression, generation of rat monoclonal antibodies, immunocytochemistry, immunohistochemistry, Western blot and FACS analyses are outlined in the Appendix S1.

## Funding

The Brain Donation Program is supported by the National Institute on Aging [P30 AG19610 Arizona Alzheimer's Disease Core Center], the Arizona Department of Health Services [contract 211002, Arizona Alzheimer's Research Center], the Arizona Biomedical Research Commission [contracts 4001, 0011, 05‐901 and 1001 to the Arizona Parkinson's Disease Consortium] and the Michael J. Fox Foundation for Parkinson's Research. This work was supported by the Carl‐von‐Linde‐Junior fellowship of the Institute for Advanced Study, Technical University Munich (PHK), the German Research Foundation RO 2226/13‐1 to SR, the Alzheimer Forschungsinitiative e.V. (AFI #11861) to MM, the Alzheimer research award of the Breuer Foundation (to SFL), an IWT grant (to SFL), the JPND Programme RiModFTD (to SFL) and the JPND Programme CrossSeeds (#01ED1501B to SR).

## Author contributions

PHK, CH, MM, CJ, ERZ, M‐GDS, GF, AS, SR and UZ designed and performed experiments. PHK, CH, AS, MM, SR, SvH, UZ and SFL analysed data. SvH, FC and EK provided research tools. PHK, SR and SFL wrote the manuscript. All authors read and approved the manuscript.

## Conflict of interest

None declared.

## Supporting information


**Fig. S1** Dilution curve of 1D1 and 6E10 antibodies with indicated dilutions on 18‐month‐old wild‐type and Tg2576 mice to investigate the specificity of 6E10 for hAPP.
**Fig. S2** Transgenic hAPP expression during aging of Tg2576 mice between postnatal month 3 and 20.
**Fig. S3** Induction of hAPP expression after traumatic brain injury.
**Appendix S1** Methods.
