# Supplementary material for: Differential transgene expression patterns in Alzheimer mouse models revealed by novel human amyloid precursor protein‐specific antibodies
Source: Aging Cell. 2016 Jul 29;15(5):953–63. doi: 10.1111/acel.12508 (PMC5013031; doi:10.1111/acel.12508)
Supplement: Supplementary file 1 — Fig. S1 Dilution curve of 1D1 and 6E10 antibodies with indicated dilutions on 18‐month‐old wild‐type and Tg2576 mice to investigate the specificity of 6E10 for hAPP. Fig. S2 Transgenic hAPP expression during aging of Tg2576 mice between postnatal month 3 and 20. Fig. S3 Induction of hAPP expression after traumatic brain injury. Appendix S1 Methods. [file ACEL-15-953-s001.pdf]

Supplementary Information to

Differential transgene expression patterns in Alzheimer mouse models revealed by novel human APP-specific antibodies

Corinna Höfling, Markus Morawski, Ulrike Zeitschel, Elisa R. Zanier, Katrin Moschke, Alperen Serdaroglu, Fabio Canneva, Stephan von Hörsten, Maria-Grazia De Simoni, Gianluigi Forloni, Carsten Jäger, Elisabeth Kremmer, Steffen Roßner, Stefan F. Lichtenthaler, Peer-Hendrik Kuhn

Supplementary Information 1

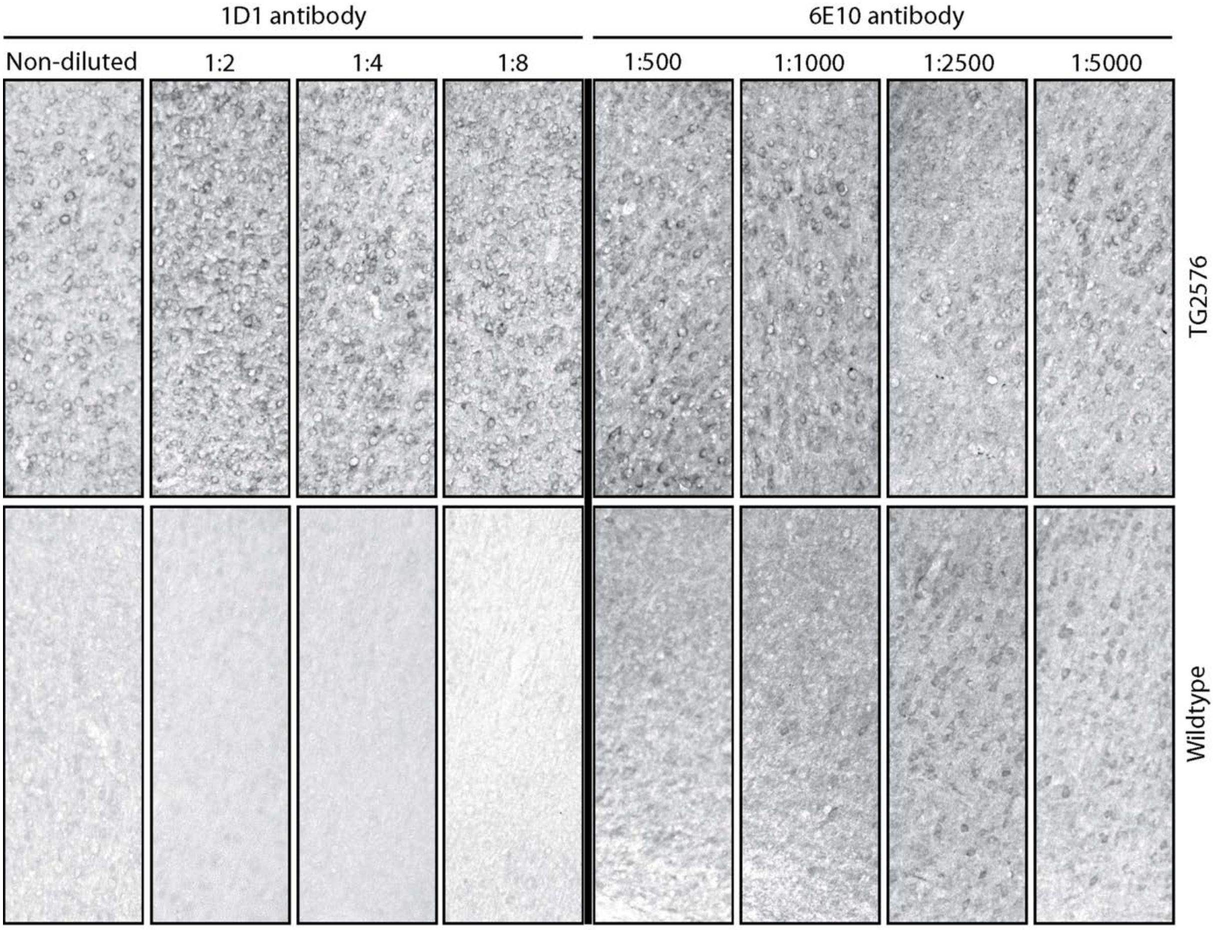

Supplementary Figure 1: Dilution curve of 1D1 and 6E10 antibodies with indicated dilutions on 18 month old wild type and Tg2576 mice to investigate the specificity of 6E10 for hAPP. At low dilutions (2 µg/ml to 1 µg/ml) 6E10 staining resulted in a strong background labelling in wild type mice. At higher dilutions we obtained a neuronal staining. In transgenic mice 6E10 produced a neuronal staining with a decrease in background staining upon an increase of the antibody dilution. 1D1 staining yielded no background staining in wild type mice irrespective of the dilution and gave a clear staining in Tg2576 mice at all dilutions.

Supplementary Information 2

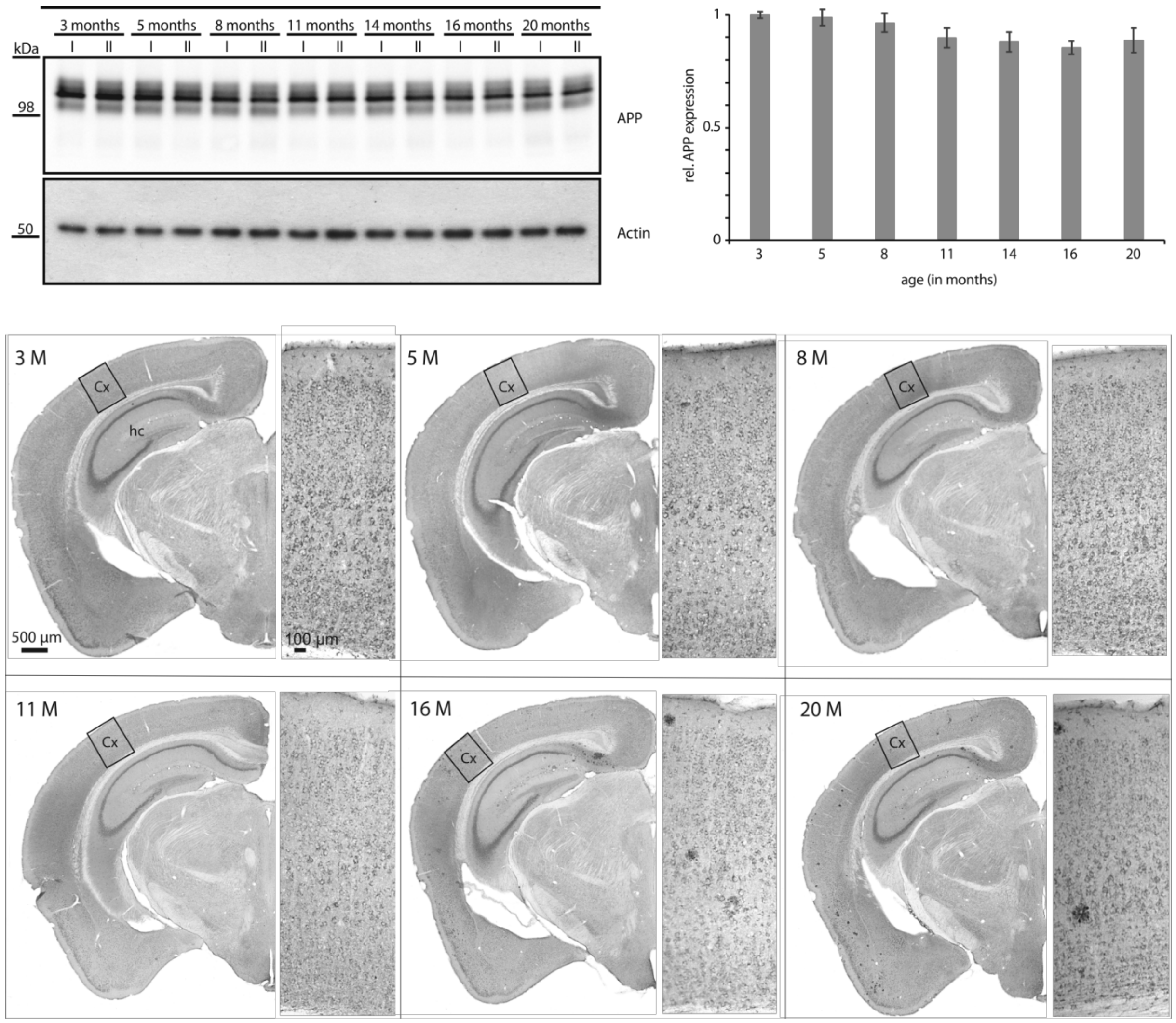

Supplementary Figure 2: The transgenic hAPP expression was investigated during aging of Tg2576 mice between postnatal month 3 and 20 by Western blot analysis and by immunohistochemistry. Western blot analysis using 2 biological replicates was performed using the antibody 1D1 in the Triton-X100 fraction of mouse brain homogenates from mice at 3, 5, 8, 11, 14, 16 and 20 months of age (top left). After normalization for actin content, the relative hAPP expression levels were quantified (top right). There were no statistically significant changes between any of the ages analyzed. Immunohistochemistry revealed similar staining pattern in brain and the labelling of the same neuronal populations as described in Fig. 2D of the main manuscript. Neocortical Abeta deposits were detected from 11 months onwards.

Supplementary Information 3

Animals

APP23 transgenic mice, overexpressing the human amyloid-β protein precursor, were generated by pronuclear insertion of APP751 cDNA with the Swedish double mutation (K670N/-M671L) into an expression cassette comprising the murine Thy-1.2 gene. The founder mice were from Novartis and back-crossed in-house with C57BL/6J (Balducci et al., 2010).

Mice were housed in a specific pathogen free vivarium at a constant temperature (21±1°C) with a 12h light–dark cycle and ad libitum access to food and water. The IRCCS-Istituto di Ricerche Farmacologiche Mario Negri (IRFMN) adheres to the principles set out in the following laws, regulations, and policies governing the care and use of laboratory animals: Italian Governing Law (D.lgs 26/2014; Authorization n.19/2008-A issued March 6, 2008 by Ministry of Health); Mario Negri Institutional Regulations and Policies providing internal authorization for persons conducting animal experiments (Quality Management System Certificate – UNI EN ISO 9001:2008 – Reg. N° 6121); NIH Guide for the Care and Use of Laboratory Animals (2011 edition) and EU directives and guidelines (EEC Council Directive 2010/63/UE). The Statement of Compliance (Assurance) with the Public Health Service (PHS) Policy on Human Care and Use of Laboratory Animals has been recently reviewed (9/9/2014) and will expire on September 30, 2019 (Animal Welfare Assurance #A5023-01). All efforts were made to minimize animal suffering and to reduce the number of animals used.

Experimental Traumatic Brain Injury

Anesthetized mice (isoflurane: 3% - induction, 1.5% - maintenance) were placed in a stereotaxic frame and subjected to craniectomy followed by controlled cortical impact brain injury as previously described (Pischiutta et al., 2014; Zanier et al., 2011). Briefly, a 3-mm rigid impactor driven by a pneumatic piston and rigidly mounted at an angle of 20° from the vertical plane was applied perpendicularly to the exposed dura mater over the left parietotemporal cortex (antero-posteriority: – 2.5 mm, laterality: – 2.5 mm) at impactor velocity of 5m/s and deformation depth of 1mm. The craniotomy was then covered with a cranioplasty and the scalp sutured. Mice were kept at 37°C throughout the duration of the surgery.

Brain transcardial perfusion

Twenty four hours post-injury, mice were deeply anesthetized (ketamine 30 mg/medetomidine 0.3 mg), and transcardially perfused with 20 ml of PBS, 0.1 mol/liter, pH 7.4, followed by 50 ml of chilled paraformaldehyde (4%) in PBS. The brains were carefully removed from the skull and post fixed for 6h at 4°C, and then transferred to 30% sucrose in 0.1 mol/L phosphate buffer for 24h until equilibration (Zanier et al., 2015). The brains were frozen by immersion in isopentane at -45°C for 3 minutes before being sealed into vials and stored at -80°C until use.

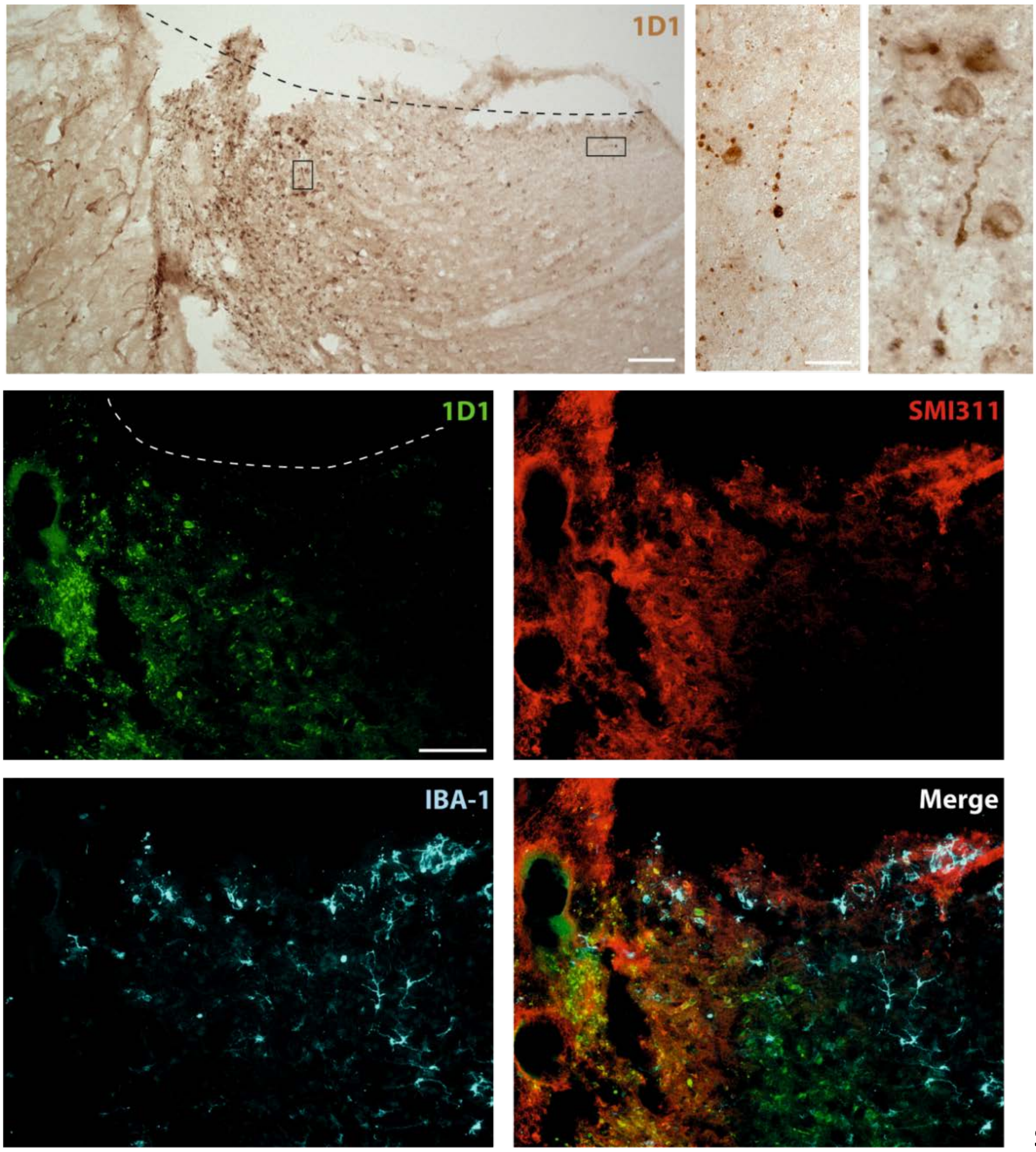

Supplementary Figure 3: The labelling of brain sections at 24 hours after traumatic brain injury following immunohistochemical protocols as outlined in the main text revealed the induction of hAPP transgene expression in neurons of the border zone of the injury (top images). In particular, swollen and fragmented neuronal processes were evident (top row, high magnification images). Triple immunofluorescent labelling of hAPP by 1D1 with the neurofilament marker SMI311 and the microglial marker IBA-1 demonstrate a complementary labelling of microglia in the injury core and of hAPP in neurons in the lesioned border zone. Scale bars in overview images represent 100 µm; Scale bar in high magnification image represents 10 µm.

References

Balducci, C., Tonini, R., Zianni, E., Nazzaro, C., Fiordaliso, F., Salio, M., Vismara, L., Gardoni, F., Di Luca, M., Carli, M., et al. 2010. Cognitive deficits associated with alteration of synaptic metaplasticity precede plaque deposition in AβPP23 transgenic mice. J. Alzheimers Dis. 21, 1367–1381.

Pischiutta, F., D'Amico, G., Dander, E., Biondi, A., Biagi, E., Citerio, G., De Simoni, M.-G., and Zanier, E.R. 2014. Immunosuppression does not affect human bone marrow mesenchymal stromal cell efficacy after transplantation in traumatized mice brain. Neuropharmacology 79, 119–126.

Zanier, E.R., Montinaro, M., Vigano, M., Villa, P., Fumagalli, S., Pischiutta, F., Longhi, L., Leoni, M.L., Rebulla, P., Stocchetti, N., et al. 2011. Human umbilical cord blood mesenchymal stem cells protect mice brain after trauma. Crit. Care Med. 39, 2501–2510.

Supplementary methods

Cloning

A cDNA encoding the hAPP ectodomain (1-596) was subcloned into pcDNA3.1/Zeo(+)-Linker-Precission-StrepII vector with HindIII/XbaI to generate pcDNA3.1-hAPP-Linker-Precission-StrepII. hAPP695 full length was subcloned into FU vector with NheI/Not to generate FU-APP. plKO2mod-EGFP-WPRE-hsAPP-2603 knockdown vector was generated cloning an oligo duplex coding for a target sequence in the hAPP 3'UTR (ccttagccagttgtatattat) between the MluI/XmaI site of plKO2mod-EGFP-WPRE vector. hAPP695 in pCR8 was subcloned into FKP/UAS-GW(DEST) via recombination mediated cassette exchange with the gateway system to obtain FKP/UAS-APP. Gal4-VP16 was subcloned into FhSyn with BamH1/Not1 to obtain FhSyn-Gal4-VP16. Construction of APP chimeras was performed amplifying the very same fragments from human and mouse APP to subsequently combine and assemble them in a second PCR to a full APP sequence. The following primers were used: APP chim 01 fw: GTCTACCCTGAACTGCAGATCAC, APP chim 01 rev: GTGATCTGCAGTTCAGGGTAGAC APP chim 02 fw: ATCTTCACTGGCACACCGTCG, APP chim 02 rev: CGACGGTGTGCCAGTGAAGAT, APP chim 03 fw: CATGCCCATTTC CAGAAAGCCAAAG, APP chim 03 rev: CTTTGGCTTTCTGGAAATGGGCATG.

hAPP antigen production

Stable hAPP-Linker-StrepII expressing pools of HEK293T cells were selected with 200 µg/ml Zeocin. For antigen production 150 ml DMEM/10% FCS were conditioned in one confluent triple layer flask for 48 hours. Conditioned medium (300 ml) was filtered with Steritop 0.45 µm filter unit (Millipore) and hAPP-linker-StrepII was subsequently purified with 300 µl of Streptactin sepharose (IBA GmbH) in a polyprep column (Biorad) from the clarified medium according to the instructions of the manufacturer. After purification 1.5 mg of pure hAPP ectodomain was obtained.

Generation of rat monoclonal antibodies against hAPP ectodomain

50 µg of the purified hAPP-StrepII fusion protein (hAPP) were injected intraperitoneally (i.p.) and subcutaneously (s.c.) into LOU/C rats using incomplete Freund's adjuvant supplemented with 5 nmol

CpG 2006 (TIB MOLBIOL, Berlin, Germany). After a six weeks interval a final boost with 50 µg hAPP and CpG 2006 was given i.p. and s.c. three days before fusion. Fusion of the myeloma cell line P3X63-Ag8.653 with the rat immune spleen cells was performed according to standard procedures. Hybridoma supernatants were tested in a solid-phase immunoassay with hAPP or an irrelevant StrepII fusion protein coated to ELISA plates. Antibodies from tissue culture supernatant bound to hAPP were detected with HRP conjugated mAbs against the rat IgG isotypes (TIB173 IgG2a, TIB174 IgG2b, TIB170 IgG1 all from ATCC, R-2c IgG2c homemade), thus avoiding mAbs of IgM class. HRP was visualized with ready to use TMB (1-Step^{TM} Ultra TMB-ELISA, Thermo). MAbs that reacted specifically with the hAPP were further analysed in non-reducing Western blot and immunocytochemistry. hAPP 1D1 and 7H6, both of rat IgG1 subclass were used in this study.

Immunocytochemistry

Neurons or HEK293T cells were fixed with 4% PFA for 10 min at room temperature, permeabilised with 0.1% TritonX-100 for 2 min and then blocked for 15 min with 5% (w/v) BSA in PBS. 1D1 and 7H6 hybridoma supernatants were diluted 1:50 in 5% (w/v) BSA in PBS and cells were stained for 1 hour on ice. Afterwards cells were washed once with PBS and stained with anti-rat Alexa Fluor 555 diluted 1:1000 in 5% (w/v) BSA in PBS and investigated under a LEICA confocal microscope.

Immunohistochemistry

Immunohistochemistry for hAPP was performed on mouse brain sections of wild type, I5, Tg2576, 3xTg mice using the mouse monoclonal antibody 22C11 raised against an N-terminal APP epitope (5 µg/ml) or the novel rat monoclonal antibodies 1D1 and 7H6 against the N-terminus of hAPP (15 µg/ml) following standard protocols using secondary biotinylated goat anti-mouse or anti-rat antibodies (Dianova; 1:400) and the ABC method. The hAPP immunoreaction was visualized by incubation with 4 mg 3,3'-diaminobenzidine (DAB) and 2.5 µl H_2O_2 per 5 ml Tris buffer (0.05 M, pH 7.6) for 1-2 minutes.

In order to reveal the cell type-specific labelling generated by the rat anti-APP antibody 1D1 double immunofluorescent labelling in combination with monoclonal antibodies from mouse directed against

the NeuN antigen was performed. Additionally, the staining pattern of 1D1 was compared to that of 22C11 by double labelling. The composition of Abeta plaques was analysed by combining the rat anti hAPP antibody 1D1 with the mouse anti-APP antibody 22C11, the mouse anti-Abeta antibodies 6E10 and 4G8 and with thioflavin S. Brain sections were incubated with cocktails of primary antibodies overnight at 4°C. On the next day, sections were washed three times with TBS and were then incubated with biotinylated goat anti-rat, 1:400 and Cy2-conjugated goat anti-mouse, 1:200, followed by Cy3-conjugated streptavidin 1:100 (Dianova) for 60 minutes at room temperature. Confocal images were taken using the LSM 510 (Zeiss, Oberkochen, Germany) microscope.

FACS

Living cells were detached in PBS with 2 mM EDTA and subsequently stained with 7H6 hybridoma supernatant diluted 1:50 in 5% (w/v) BSA in PBS for 1 hour on ice. Afterwards cells were washed once with PBS and stained with anti-rat Alexa Fluor 555 diluted 1:1,000 in 5% (w/v) BSA in PBS for 1 hour on ice. Finally cells were washed twice with 5% (w/v) BSA in PBS. Finally cells were analyzed with a FACS Canto III (BD Bioscience). Resulting data were analyzed with FlowJo.

Brain tissue homogenates

PBS soluble fractions were prepared from neocortex of 18 months old mouse and rat as well as human brain samples (AD case: male, 77 years; control case: male, 82 years) in PBS containing complete protease inhibitor cocktail (Roche) followed by a clarifying centrifugation step at 15,000 g. The remaining pellet was solubilized in 1% Triton in STE buffer (150 mM NaCl, 50 mM Tris, 2 mM EDTA) followed by a clarifying spin at 15,000 g. Samples were used for non-reducing and reducing SDS PAGE and 20 µg total protein of mouse, rat and human brain homogenates were loaded onto 10% polyacrylamide gels.

Western Blot

Samples were boiled for 5 min at 95°C in non-reducing Laemmli buffer for detection with the anti-hAPP antibodies 7H6 and 1D1 which only work under non-reducing conditions. For detection with 22C11 samples were boiled in reducing Laemmli buffer. SDS page and transfer were carried out using exclusively nitrocellulose membranes. The highest signals were detected for Tg2576 mouse brain and the amount of protein loaded was reduced to 15 µg per lane to allow the use of similar detection conditions. The membranes were blotted overnight and incubated with 22C11 (1 µg/ml) and 1D1 (3 µg/ml) antibodies for 2 hours at room temperature, followed by incubation with secondary, horseradish peroxidase-conjugated goat anti-mouse and goat anti-rat antibodies and chemiluminescent visualisation.
